# A comparison of covariate adjustment approaches under model misspecification in individually randomized trials

**DOI:** 10.1186/s13063-022-06967-6

**Published:** 2023-01-06

**Authors:** Mia S. Tackney, Tim Morris, Ian White, Clemence Leyrat, Karla Diaz-Ordaz, Elizabeth Williamson

**Affiliations:** 1 Department of Medical Statistics, London School of Hygiene and Tropical Medicine, London, UK; 2 MRC Biostatistics Unit, University of Cambridge, Cambridge, United Kingdom; 3 MRC Clinical Trials Unit at UCL, London, UK; 4 Department of Statistical Science, UCL, London, United Kingdom

**Keywords:** Covariate adjustment, Randomized controlled trials, Misspecification, ANCOVA, G-computation, IPTW, AIPTW, TMLE

## Abstract

Adjustment for baseline covariates in randomized trials has been shown to lead to gains in power and can protect against chance imbalances in covariates. For continuous covariates, there is a risk that the the form of the relationship between the covariate and outcome is misspecified when taking an adjusted approach. Using a simulation study focusing on individually randomized trials with small sample sizes, we explore whether a range of adjustment methods are robust to misspecification, either in the covariate–outcome relationship or through an omitted covariate–treatment interaction. Specifically, we aim to identify potential settings where G-computation, inverse probability of treatment weighting (IPTW), augmented inverse probability of treatment weighting (AIPTW) and targeted maximum likelihood estimation (TMLE) offer improvement over the commonly used analysis of covariance (ANCOVA). Our simulations show that all adjustment methods are generally robust to model misspecification if adjusting for a few covariates, sample size is 100 or larger, and there are no covariate–treatment interactions. When there is a non-linear interaction of treatment with a skewed covariate and sample size is small, all adjustment methods can suffer from bias; however, methods that allow for interactions (such as G-computation with interaction and IPTW) show improved results compared to ANCOVA. When there are a high number of covariates to adjust for, ANCOVA retains good properties while other methods suffer from under- or over-coverage. An outstanding issue for G-computation, IPTW and AIPTW in small samples is that standard errors are underestimated; they should be used with caution without the availability of small-sample corrections, development of which is needed. These findings are relevant for covariate adjustment in interim analyses of larger trials.

**Supplementary Information:**

The online version contains supplementary material available at 10.1186/s13063-022-06967-6.

## Background

Whether to adjust for baseline covariates in the analysis of randomized clinical trials is a question that has attracted controversy. In trials, the aim is to estimate the marginal effect of the treatment. While unadjusted analyses in individually randomized trials are unbiased on average, there are several reasons why covariate adjusted approaches are attractive. Firstly, if covariates are used in the randomization procedure by, for example, permuted blocks or minimization, it is necessary to adjust for the covariates [1]. Secondly, adjusting for covariates that are not used for randomization can lead to statistical advantages. Adjustment for covariates that are correlated with the outcome (prognostic covariates), such as the outcome measured at baseline, typically leads to increases in power. Kahan et al. [2] showed that adjustment for prognostic covariates leads to substantial increases in power for moderate to large trials for continuous, binary and time-to-event outcomes. Covariate adjustment can offer protection against chance imbalance in the distribution of the covariates between treatment groups, which is particularly relevant for smaller trials [3]. Guidelines for clinical trials typically mention these potential benefits and caution against “fishing” for covariates that impact the statistical significance of the treatment effect [4].

Researchers have also addressed the topic of covariate adjustment from a finite population perspective, finding that concerns raised about the possibility of covariate adjustment decreasing precision [5] were largely resolved in large samples [6]. In the current manuscript, we instead take the perspective of an infinite super-population from which we consider our trial population to be drawn.

Covariate adjustment is often achieved by a regression approach modelling the effects of the treatment and covariates. We refer to this in the continuous outcome case as the analysis of covariance (ANCOVA) and in the binary outcome case as direct regression adjustment, which we abbreviate to *direct RA*. The marginal treatment effect of interest may be a parameter of the model, or it may be a derived quantity of the model. For estimands that are collapsible, such as the difference in means for a continuous outcome or the risk difference for a binary outcome, the marginal effect of treatment is a parameter of the model if there are no covariate–treatment interactions. For non-collapsible estimands such as the odds ratio for a binary outcome, adjusting for covariates changes the estimand for parameters directly estimated by the model [7, 8], so the marginal treatment effect must be a derived quantity. In the special case where treatment–covariate interactions exist, a regression-based approach does not allow the direct estimation of the marginal effect, so the marginal effect is a derived quantity.

Practitioners may be reluctant to adopt a covariate-adjusted approach [1], due to the potential for mis-specifying the model relating the outcome, treatment and covariates. This issue is particularly pronounced when covariates are continuous, since the functional form of the relationship between the covariate and outcome needs to be specified. In addition to concerns around non-collapsibility of the estimand, misspecification of this functional form could potentially lead to reduced power and could also lead to bias for continuous outcomes where sample size is small [9]. There may also be reluctance to adopt an adjusted approach in smaller trials due to the loss in degrees of freedom. These concerns may also lead to reluctance in taking a covariate adjusted approach in interim analyses of larger trials. The European Medicines Agency [10] recommend using a simple functional form (e.g. linear or categorization) if the relationship between a continuous covariate and outcome is unknown, and discourage the inclusion of covariate–treatment interactions. Recent draft guidelines from the Food and Drug Administration [11] suggest that interactions may be included, but the primary analysis should still estimate the average treatment effect. Kahan et al. [12] studied the impact of several adjustment methods, including categorization of continuous variables, modelling the effect of the covariate with a linear effect, with fractional polynomials and cubic splines. They investigated the effect on power, bias and type I error of moderate to large trials ($n=200$ to 600). Their recommendation is to use fractional polynomials or restricted cubic splines.

In addition to ANCOVA, we consider covariate adjustment methods that are less commonly used in the analysis of randomized trials: G-computation, also known as *standardization* or *marginalization*, which requires a model for the covariate–outcome relationship but targets the marginal estimand; inverse probability of treatment weighting (IPTW), which does not require modelling of the covariate–outcome relationship but instead models the treatment allocation mechanism in order to balance covariates between arms; and two approaches, augmented inverse probability of treatment weighting (AIPTW) and targeted maximum likelihood estimation (TMLE), which involve specification of both types of models but require only one to be consistently estimated. G-computation was used for covariate adjustment in a trial investigating antiretroviral treatment with standard care [13]. IPTW as a covariate adjustment approach has been demonstrated in re-analyses of trials [14–16].

In randomized trials, both unadjusted and a range of adjusted estimators of marginal treatment effect can be shown to belong to a class of methods which produce consistent and asymptotically normal treatment effect estimators, irrespective of whether the covariate adjustment is correctly specified [17, 18]. White et al. [19] cautioned against using non-canonical link functions (as might be done to estimate a non-standard marginal estimand in a direct regression approach) as it can lead to bias under the null hypothesis. While there are a range of estimators that are protected against the risks of misspecification in sufficiently large samples, the properties of adjustment methods in small trials have received limited attention.

In this study, we focus on the question of whether adjusting for continuous baseline covariates is beneficial in smaller sized trials where there is risk of misspecification of the covariate–outcome relationship. We consider the specific case of a trial with a binary treatment where randomization is 1:1 on the individual level (no blocking/stratification is used), and the marginal treatment effect is of interest. We use a simulation study to explore the extent to which the known benefits of adjustment in large trials—gain in power while estimates remain unbiased and coverage remains at the nominal level—are retained in smaller-sized trials in the presence of model misspecification. In particular, we wish to identify whether any of the lesser-known adjustment approaches offer improvement over the commonly used ANCOVA. As this study is designed to identify corner cases that tease out differences between these related approaches, our simulation study explores a number of extreme settings that are unlikely to be encountered in practice, but can provide insight into the properties of these methods.

## Methods

We consider continuous or binary outcomes, *Y*. We denote the potential outcome when a participant is given treatment *z* by $Y^z$, where $z=0$ is the control and $z=1$ is the active treatment. We denote a baseline covariate by *X*. For a continuous outcome, the marginal treatment effect is defined by taking the difference between the marginal mean of the outcomes under the active treatment, and the marginal mean of the outcomes under the control:

1 $$\begin{aligned} \mathbb {E}(Y^1)-\mathbb {E}(Y^0). \end{aligned}$$

For a binary outcome, we consider two estimands of interest, the risk difference (RD):

2 $$\begin{aligned} \mathbb {P}(Y^1=1)-\mathbb {P}(Y^0=1), \end{aligned}$$

and the marginal odds ratio (OR),

3 $$\begin{aligned} \frac{\mathbb {P}(Y^1=1)/\mathbb {P}(Y^1=0)}{\mathbb {P}(Y^0=1)/\mathbb {P}(Y^0=0)}. \end{aligned}$$

For a continuous outcome, an unadjusted analysis involves fitting the following model, and taking the estimated coefficient $\hat{\beta }$ as the treatment effect estimate, which is the difference between the sample mean of the outcomes under the active treatment and the sample mean of the outcome under the control:

4 $$\begin{aligned} \mathbb {E}\left( Y \mid Z \right) =\alpha +\beta Z. \end{aligned}$$

For a binary outcome, we consider two unadjusted models. Firstly, a binomial model with an identity link function to estimate the risk, where the left-hand side of Eq. (4) is $\mathbb {P}(Y=1 \mid Z)$, then the coefficient $\beta$ is the risk difference. Secondly, a binomial model with a logit link function can be used to estimate the log-odds, where the left-hand side of Eq. (4) is $\log \frac{\mathbb {P}(Y=1)}{\mathbb {P}(Y=0)}$ and ${\beta }$ represents the marginal log odds ratio.

### Regression approaches

The most common approach to covariate adjustment in trials is through an analysis of covariance (ANCOVA), where the expectation of the outcome given the treatment and covariate is specified by a linear model:

5 $$\begin{aligned} \mathbb {E}(Y \mid Z, X) = \alpha + \beta _x Z + \gamma X. \end{aligned}$$

The treatment effect estimate is given by $\hat{\beta }_x$, where the subscript emphasizes that the coefficient for treatment is adjusted for the covariate value *x*. This model can be extended to include additional covariates and/or non-linear functions of covariates, in which case $\varvec{X}$ is a vector including functions of the covariate values. The ANCOVA treatment estimate has very desirable robustness properties in large samples; it is consistent [9], and its standard error is consistent where randomization is 1:1, even when the model is misspecified [18].

The are two ways in which the adjusted model in Eq. (5) could be misspecified. Firstly, ANCOVA models the relationship between the covariate and outcome as linear; in other words, the effect of a one-unit increase in the covariate on the outcome is constant for all values across the range of the covariate. The true underlying covariate–outcome relationship could be a more complex non-linear relationship. To address this issue of potential non-linearity, the model can be adapted to allow a more flexible specification involving splines, which can capture non-linearities in the covariate–outcome relationship. The range of the covariate is split into *m* sections and, within each section, the covariate–outcome relationship is specified by a cubic polynomial. The $m-1$ resulting curves are joined at knots to create a smooth function. The addition of splines leads to an additional $m+1$ degrees of freedom required to fit the model. In this study, we place knots at equally spaced quantiles of the covariate. Secondly, there may be interactions between the treatment and covariate that are not reflected in the model. While ANCOVA will lead to consistent estimators even if the model is misspecified in large samples [4], the properties of estimators for smaller sample sizes are less known.

For a binary outcome, an analogous adjusted model can be specified for the risk or log-odds. As discussed earlier, covariate adjustment changes the estimand in the case of the odds ratio, so an adjusted regression model may not be a pragmatic approach. For further discussion of estimands for binary outcomes using the counterfactual framework, see, for example, Didelez and Stensrud [20] or  Daniel et al [8]. With the risk difference as the estimand, smaller sample sizes can lead to well-known convergence problems [21].

### G-computation

G-computation is a standardization approach which can be used to obtain an adjusted estimate of the marginal treatment effect. A model for the mean outcome in terms of *Z* and *Y* is specified:

6 $$\begin{aligned} m(Z, X) = \mathbb {E}(Y \mid Z, X), \end{aligned}$$

and used to predict the expected value of both potential outcomes for each individual. The mean outcome $\mathbb {E}(Y^1)$, under a possibly counterfactual assignment to treatment, is then estimated by the sample average of the predicted outcomes $\hat{Y}^1$, and analogously, the mean outcome under the control arm $\mathbb {E}(Y^0)$ is computed:

7 $$\begin{aligned} \widehat{\mathbb {E}}(Y^z) = \frac{1}{n} \sum \limits _{i=1}^n \hat{m}(Z_i=z, x_i). \end{aligned}$$

The treatment effect estimate is the difference between the estimated mean outcomes under the two treatments. If *m*(*Z*, *X*) is Eq. (5), the resulting treatment effect estimate is equal to the ANCOVA estimate. However, the covariate–outcome relationship can be modelled separately in each treatment group, which is equivalent to including a main effect and interaction between the treatment and covariate in Eq. (5), and marginalizing (as described above) to obtain an overall estimate of treatment effect. A nonlinear covariate–outcome relationship could also be specified, for example by the use of splines. An advantage of this approach is that it separates the final estimation of the treatment effect from the modelling of the outcome.

For binary outcomes, a binomial model with logit link can be used to predict the potential outcomes on the probability scale. The sample averages can be attained to estimate $\mathbb {P}(Y^1=1)$ and $\mathbb {P}(Y^0=1)$, and the odds ratio or risk difference can be computed. There are particular advantages to using G-computation for the binary outcome case. Firstly, if the summary measure of interest is the risk difference, convergence problems that affect direct regression approaches can be avoided. Secondly, if the odds ratio is the estimand of interest, G-computation achieves covariate adjustment while retaining the marginal estimand; the issue of adjustment changing the estimand is avoided.

G-computation can be written as an M-estimator, which relies on large-sample approximations to derive standard errors and confidence intervals [22]. The standard errors are underestimated when sample sizes are small [23], which translate to undercoverage and false gains in power. Bartlett [24] showed that the estimates of marginal means $\mathbb {E}(Y^z)$ are consistent for canonical generalized linear models, even if the model is misspecified. Therefore, in large samples, we expect the difference in marginal means (for the continuous outcome case) and the risk difference, marginal odds ratio and relative risk (in the binary outcome case) to be consistently estimated, even if the model is misspecified.

### IPTW

Propensity score-based methods have largely been used in observational studies to address confounding and selection bias; however, Williamson et al. [14] demonstrated they lead to similar large-sample properties as ANCOVA, such as increases in power, when applied to randomized controlled trials. Inverse probability of treatment weighting (IPTW) involves specifying a model for the propensity score, which is the probability that a participant is assigned the active treatment, given values of their covariates: $e(X) = P(Z=1 | X).$ It may seem counter-intuitive to estimate the propensity score in a simple trial setting, since randomization implies that the true propensity score is 0.5. However, chance imbalance of covariates will be reflected in estimated propensity scores, which can then be re-balanced using a weighting approach. The propensity score can be estimated using logistic regression, by modelling *Z* as a binomial distribution, where:

8 $$\begin{aligned} \text {logit} \left\{ e(X) \right\} = \delta + \kappa X. \end{aligned}$$

Outcomes for participants that received the active treatment are weighted by $\frac{1}{\hat{e}(x_i)}$, and outcomes for participants that received the placebo are weighted by $\frac{1}{1-\hat{e}(x_i)}$. The estimated weighted mean is given by:

9 $$\begin{aligned} \widehat{\mathbb {E}}(Y^z) = \frac{1}{n} \sum \limits _{i=1}^n \frac{y_i \mathbb {I}(Z_i=z)}{\hat{e}(x_i)^{z_i}\left( 1-\hat{e}\left( x_i\right) \right) ^{(1-z_i)}}, \end{aligned}$$

and the treatment effect estimate is the difference between the estimated weighted mean outcomes under the two treatments. We note that this is fitting a model for the mean outcome, such as in Eq. (4), where the outcomes are weighted by the inverse probability of being assigned the arm that they were randomized to. The regression approach can more easily be adapted to provide valid standard errors. For binary outcomes, a binomial model is specified for the mean outcome instead, with a linear link function for the risk difference, or a logistic link function for the marginal odds ratio.

A major attraction of this approach is that it avoids modelling the covariate–outcome relationship, and the potential for covariate–treatment interactions does not need to be considered. In certain settings, for example in 1:1 randomization, the propensity score is correctly specified. Further, and similarly to G-computation, a feature of IPTW for binary outcomes is that the marginal estimand for the odds ratio is estimated. IPTW also belongs to the class of M-estimators whose variance estimators rely on large-sample properties [22] which have been found to perform poorly in some small sample settings [15].

### AIPTW and TMLE

Finally, we consider two approaches, augmented inverse probability-of-treatment weighting (AIPTW) and targeted maximum likelihood estimation (TMLE), that require a model for the covariate–treatment relationship as well as a model for the treatment assignment. They are known as *doubly robust* estimators as only one of the two models needs to be correctly specified to be consistent for the treatment effect [25]. In the trial setting with 1:1 randomization, since the propensity score is correctly specified, we obtain consistent estimators even if the outcome model is misspecified.

Augmented inverse probability-of-treatment weighting (AIPTW) requires a model for the mean outcome, which is then used to to obtain predictions of the potential outcomes, as in G-computation. It also requires a model for the propensity score so that inverse probability of treatment weights can be calculated. These weights are then used to add an *error-correcting* term to the G-computation estimator, which is the sum of weighted differences between the observed outcomes and predicted outcomes:

10 $$\begin{aligned} \widehat{\mathbb {E}}(Y^z) = \underbrace{\frac{1}{n}\sum _{i=1}^n \hat{m}(Z_i=z, x_i)}_\text {G-computation estimator} - \underbrace{\frac{1}{n} \sum _{i=1}^n \frac{\hat{m}(Z_=z, x_i) - y_i}{\hat{e}(x_i)^{z_i}\left( 1-\hat{e}\left( x_i\right) \right) ^{(1-z_i)}}\mathbb {I}(Z_i=z)}_\text {error-correcting term}. \end{aligned}$$

Similarly to G-computation and IPTW, AIPTW belongs to the class of M-estimators which rely on large sample properties for the variance estimator [22].

Targeted maximum likelihood estimation (TMLE) requires an initial model of the covariate–outcome relationship, which could be a regression model as in G-computation, or it could be a flexible machine learning model [25]. A model for treatment assignment, such as Eq. (8), is then specified to obtain propensity scores. The propensity scores are required to compute so-called *clever covariates* for each individual, which are then used to estimate the *fluctuation parameter* for the efficient influence function using a maximum likelihood procedure [26]. The fluctuation parameter corrects the initial estimate for $\mathbb {E}(Y \mid Z, X)$. This *targeting step* optimizes the bias-variance trade-off for the treatment effect [27]. The difference between the average of predicted potential outcomes under the treatment and the average of predicted potential outcome under the control is then computed to obtain the marginal treatment effect estimate. Standard errors can be estimated using the efficient influence function evaluated for the empirical distribution, or through non-parametric approaches such as the bootstrap [28]. TMLE is asymptotically efficient for the point estimate if both the propensity score model and the model for the outcome are correctly specified [29]. Consistency of the estimated standard error requires that both models are correctly specified [30].

**Fig. 1:**
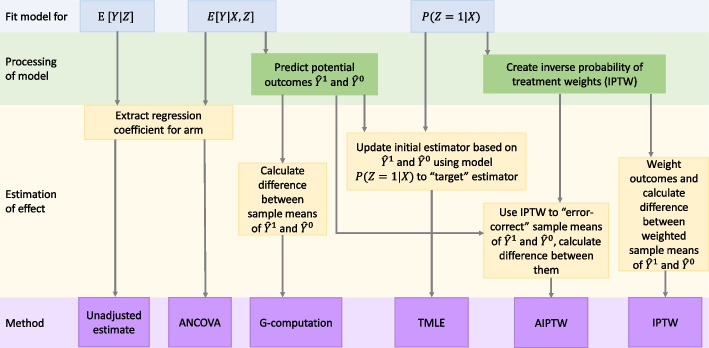
Comparison of methods for unadjusted and adjusted analyses, adapted from [27]

A comparison of the models required in these methods are illustrated in Fig. 1.

## Simulations

We performed simulation studies to compare covariate adjustment methods where the covariate–outcome model has been misspecified in small parallel design two-arm trials with 1:1 randomization. We note that our simulation settings include a number of extreme settings which are unlikely to be encountered or implemented in practice, such as the splines with 20 degrees of freedom, adjusting for 17 or more covariates, or the harmonic relationship between covariate and outcome. While unrealistic, exploring these settings allows us to pinpoint the settings where one method of covariate adjustment may offer advantages over another.

In our “Main Simulation” section, we explored the setting with one covariate, no covariate–treatment interaction, and a continuous outcome. Three smaller simulation studies vary these three design features in turn. The “Extension 1: Multiple covariates” section expands the main simulation to consider multiple covariates, with a continuous outcome and no interaction. The “Extension 2: Interaction” section adds a covariate–treatment interaction to the main simulation, with one covariate and a continuous outcome. The “Extension 3: Binary outcome” section considers the setting of a binary outcome where there is a single covariate and no interaction. In each setting, we were interested in estimating the effect of treatment and comparing the following performance measures for a number of analysis approaches:

- Bias
- Coverage of the $95\%$ confidence interval
- Model-based and empirical standard error
- Power
- Type I error rate

In each setting, total sample sizes of 25, 50 and 100 were considered where possible, and 1000 repetitions of the simulation were performed. The simulation was performed in R version 4.1.1 [31]. We provide an overview of the data generating mechanism, estimand and analysis approaches in each of the four settings. Full details of the data generating mechanisms are provided in Additional file 1, and R code is provided in Additional files 2, 3, and 4.

### Main simulation

**Fig. 2:**
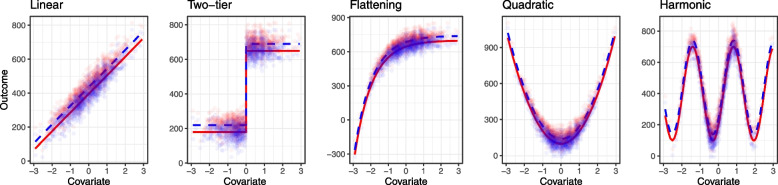
Main simulation. True relationships between the continuous outcome and treatment studied in the simulation with the dots depicting the datapoints in a single simulated dataset of 100 participants. Red indicates the active treatment and blue indicates the control

In this setting, we generated a continuous outcome from the model,

11 $$\begin{aligned} Y_i = \alpha + \beta Z_i + f(X_i) + \epsilon _i, \end{aligned}$$

where $\epsilon _i \sim N(0, 42)$, and the binary treatment $Z_i$ takes value 1 for the active arm and 0 for the placebo arm. The treatment was allocated randomly with a 0.5 probability of a participant receiving the active treatment. We considered the case with a treatment effect ($\beta =40$) and without treatment effect ($\beta =0$). The covariate is generated according to $f(X_i)$, where $X_i$ is drawn from a standard normal distribution and the function $f(\cdot )$ denotes five possible covariate–outcome relationships: *linear, two-tier, flattening, quadratic* and *harmonic*, as illustrated in Fig. 2. These relationships range from those which may plausibly be encountered in trials, through to difficult distributions unlikely to be encountered in practice, in order to explore the properties of the adjustment methods in a number of settings.

The average treatment effect, $\beta$, is the estimand of interest. We consider the following methods for estimating $\beta$:

- *Unadjusted analysis*, equivalent to a *t*-test as in Eq. (4)
- *ANCOVA* as in Eq. (5) using an *F*-test
- *G-computation*, implemented using stdGlm() in the stdReg package [32], where a single model, as in Eq. (5), is fitted to both arms
- *G-computation with interaction* where separate models assuming linear effects of covariates are fitted for each arm
- *IPTW* where the model for treatment assignment is as in Eq. (8), implemented using psw() in the PSW package [33]. The standard errors are corrected to account for the propensity score estimation
- *AIPTW*, where the model for treatment assignment is as in Eq. (8) and the model for the outcome is as in Eq. (5), implemented using psw() in the PSW package [33]
- *TMLE* where the model for treatment assignment is as in Eq. (8) and the model for the outcome is as in Eq. (5), implemented using tmle() in the TMLE package [34]. The standard error is computed using the efficient influence function evaluated for the empirical distribution

We explored the addition of splines in a selection of these methods for sample size 50 and 100: splines with 4 or 20 degrees of freedom in the regression approach, splines with 4 degrees of freedom in G-computation and splines with 4 degrees of freedom in IPTW. In all uses of splines in this study, knots are placed at equally spaced quantiles of the covariate. Splines are implemented using the ns() function in the splines package [31]. For IPTW, splines are implemented with PSweight() in the PSWeight package [35].

### Extension 1: Multiple covariates

This setting has a continuous outcome, multiple covariates and no interaction. We consider a scenario where 21 covariates are measured for each individual, of which 17 are continuous and 4 are binary. The covariates are generated to mimic typical demographic and health-related covariates in a trial setting. Briefly, 17 covariates (13 continuous and 4 binary) are predictive of the outcome, of which three continuous covariates are highly predictive of the outcome. There are four additional noise covariates. The outcome is generated with a number of linear and non-linear effects from the covariates and some covariate–covariate interactions, but no covariate–treatment interactions. We considered the case with a treatment effect ($\beta =40$) and without treatment effect ($\beta =0$). The estimand of interest is the the marginal difference in means. We explored adjusting for:

- The three highly predictive covariates only
- A larger selection of 17 potentially predictive covariates
- All 21 covariates, which include noise variables

Due to the high number of parameters in the models for adjustment, we considered sample sizes of $n=50$ and $n=100$ only. For each of the three cases, we used the following analysis methods:

- Unadjusted analysis
- ANCOVA
- G-computation
- G-computation with interaction
- IPTW
- AIPTW
- TMLE

### Extension 2: Interaction

**Fig. 3:**
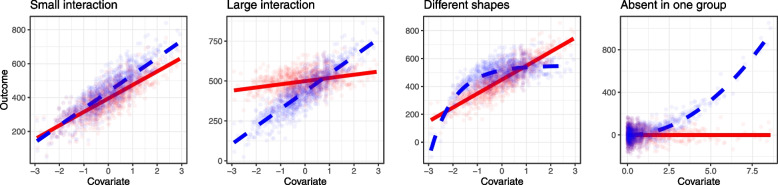
Extension 2. True relationships between the continuous covariate and outcome with interaction are shown: small interaction, large interaction, different shapes, and effect absent in one group. The dots depict the datapoints in a single simulated dataset of 100 participants, where the red dots indicate the active treatment and the blue dots indicate the control

This setting has a continuous outcome, one covariate and a covariate–treatment interaction. Four different interaction settings were considered, as illustrated in Fig. 3. A single covariate was generated from a *N*(0, 1) distribution. In the first setting, this covariate has a small interaction with the treatment. In the second setting, the covariate has a larger interaction in which the treatment effect changes direction. In the third setting, the covariate–outcome relationship has different shapes in each arm (exponential under the active treatment and linear under the placebo). Finally, in the last setting, the covariate is the square of a standard normal distribution and therefore has a skewed distribution. There is no effect of the covariate on the outcome for the active treatment, but there is an effect under the placebo. We demonstrate in the Appendix that the bias due to misspecification in a model including a covariate–treatment interaction is likely to be pronounced where there is a skewed covariate with different types of misspecification in each arm, prompting the addition of this last scenario in our simulation. This proof is the first report of this property, to our knowledge.

We consider the following methods of estimating the treatment effect:

- Unadjusted analysis
- ANCOVA
- G-computation
- G-computation with interaction
- IPTW
- AIPTW
- TMLE

### Extension 3: Binary outcome

This setting has a binary outcome, one covariate and no interaction. We generate the covariate *X* from a standard normal distribution. The outcomes are generated using Eq. 11 on the logit scale, where the function $f(\cdot )$ denotes five possible covariate–outcome relationships: *linear, two-tier, flattening, quadratic* and *harmonic*. Settings with a treatment effect (with a conditional odds ratio of 0.2) and without treatment effect were considered. Due to potential convergence issues in smaller sample sizes, we considered only the sample size $n=100$.

We considered the following estimands of interest: the risk difference, the marginal odds ratio (for all methods except direct RA with logistic link), and the data generating conditional odds ratio (for direct RA with logistic link). We consider the following methods for estimating the effect of interest:

- Unadjusted binomial regression with linear link for the risk difference or logistic link for the marginal odds ratio.
- Direct regression adjustment (RA) with logistic link for the data generating conditional odds ratio. An adjusted binomial model with a linear link for the risk difference leads to convergence issues so is omitted.
- G-computation for the risk difference or marginal odds ratio.
- IPTW for the risk difference or marginal odds ratio.
- AIPTW is included for the risk difference, but omitted for the marginal odds ratio as it is not readily available in the software used.
- TMLE for the risk difference or marginal odds ratio.

## Results

### Main simulation

**Fig. 4:**
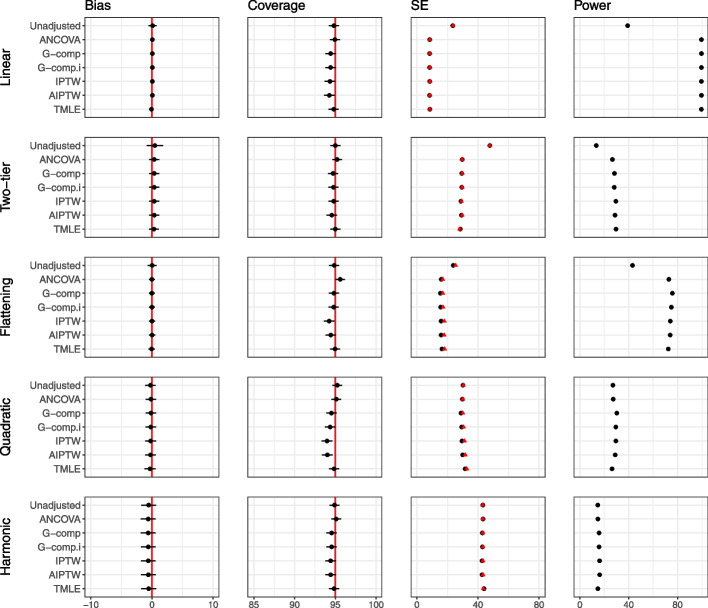
Main simulation results (continuous outcome, single covariate, no interaction) for sample size 100. The number of repetitions for this simulation is 5000. The performance of analytic methods in terms of bias, coverage and power for the five different covariate–outcome relationships is displayed. The effect of treatment is 40. Model-based standard errors are indicated in black and empirical standard errors are shown in red. Estimates are indicated with $\pm 1.96 \times$ Monte Carlo standard error bars. Note that the error bars are too small to be seen for power, due to the scale of the plots

Figure 4 displays the results for the main simulation for the analytic methods for $n=100$. In all plots in the main simulation and in the extensions, Type I error rate mirrors the coverage levels so are omitted from figures. All analytic methods considered produce unbiased estimates. We observe that the variability around the estimated effect is larger for the non-linear relationships. At this sample size, we observe that the adjustment methods generally achieve nominal coverage, except for G-computation with interaction, IPTW and AIPTW, which lead to undercoverage in the highly non-linear *quadratic* relationship. All adjustment methods produce gains in power for the *linear*, *two-tier* and *flattening* relationships. For the *quadratic* and *harmonic* relationships, which are poorly approximated by a linear relationship, adjustment methods which assume a linear covariate–outcome relationship provide no increase in power, but do not lead to any loss in power. We note that this particular simulation was run with 5000 repetitions as apparent bias purely due to chance was observed for the *two-tier* case with 1000 repetitions. This is shown in Fig. 2 in Additional file 1.

**Fig. 5:**
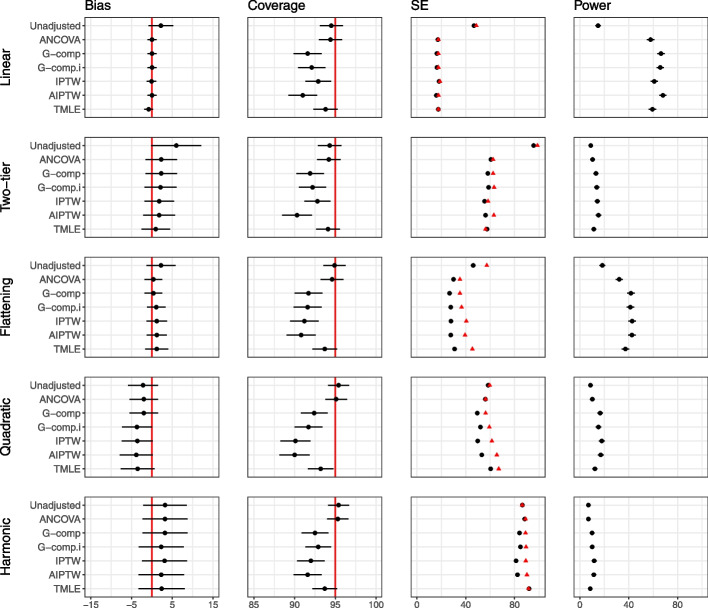
Main simulation results (continuous outcome, single covariate, no interaction) for sample size 25. The performance of analytic methods in terms of bias, coverage and power for the five different covariate–outcome relationships are displayed. The effect of treatment is 40. Model-based standard errors are indicated in black and empirical standard errors are shown in red. Estimates are indicated with $\pm 1.96 \times$ Monte Carlo standard error bars. Note that the error bars are too small to be seen for power, due to the scale of the plots

Figure 5 displays results for $n=25$, and results for $n=50$ are provided in Additional file 1. For these smaller sample sizes, we observe that standard errors produced by IPTW, G-computation and AIPTW are smaller than the empirical standard error, leading to undercoverage, high type I error and false gains in power.

**Fig. 6:**
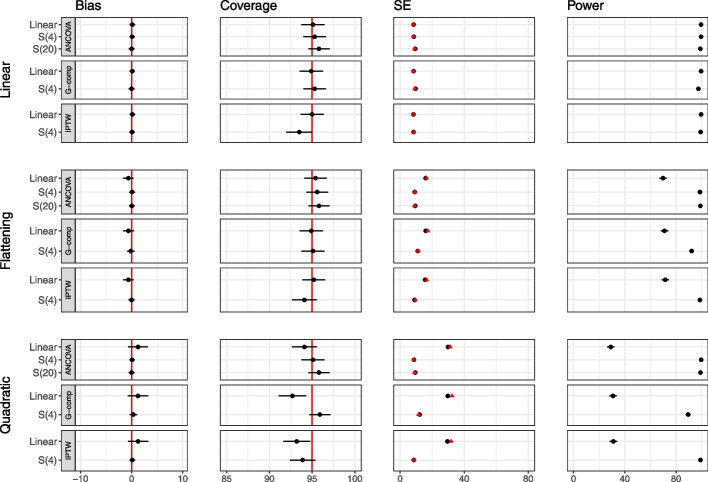
Main simulation results (continuous outcome, single covariate, no interaction) for sample size 100 for ANCOVA, G-computation and IPTW with and without the use of splines. The performance of analytic methods in terms of bias, coverage and power for the *Linear, Flattening* and *Quadratic* relationships are displayed. The effect of treatment is 40. Model-based standard errors are indicated in black and empirical standard errors are indicated in red. Estimates are shown with $\pm 1.96 \times$ Monte Carlo standard error bars. Note that the error bars are too small to be seen for power, due to the scale of the plots

In Fig. 6, the results for ANCOVA, G-computation and IPTW with and without the use of splines are shown for $n=100$ for the *linear, flattening* and *quadratic* relationships. Additional file 1 displays these results for $n=50$. We observe that the use of splines leads to considerable gains in power for the non-linear relationships, and generally does not incur bias or affect type I error. When the relationship is linear, the addition of splines generally does not affect bias, type I error or power. We observe that the discrepancy between the model-based and empirical standard errors is particularly pronounced for the IPTW approach with splines and the G-computation approach with splines when $n=50$ due to the additional parameters in the model from the splines.

### Extension 1: Multiple covariates

**Fig. 7:**
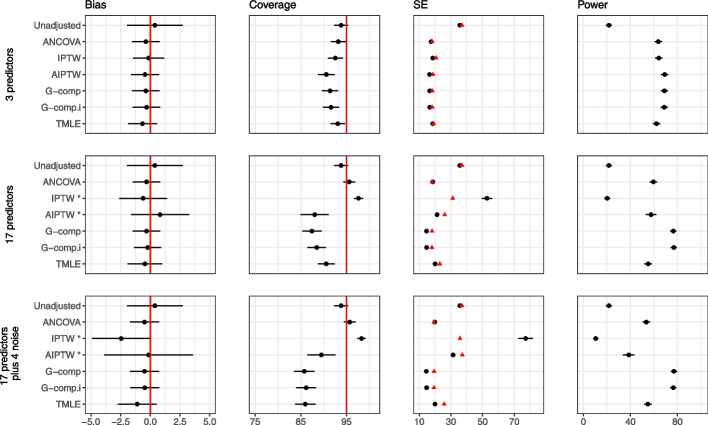
Extension 1 results (continuous outcome, multiple covariates, no interaction) for sample size 50. The performance of analytic methods in terms of bias, coverage and power are shown when there are 3 covariates, 17 covariates and 17 covariates plus 4 noise variables. The effect of treatment is 40. Model-based standard errors are shown in black and empirical standard errors are shown in red. Estimates are shown with $\pm 1.96 \times$ Monte Carlo standard error bars. Note: the error bars are too small to be seen for power, due to the scale of the plots. * AIPTW and IPTW have convergence issues when there are a high number of predictors; see Table S1 in Additional file 1 for more details

Figure 7 displays the results for the continuous outcome case with multiple covariates when $n=50$. All methods are unbiased regardless of the number of predictors, and all adjustment methods lead to gains in power when there are three predictors. When there are a large number of predictors, ANCOVA retains nominal coverage and type I error, but there are concerns with the other approaches. For IPTW, the model-based standard errors overestimate the empirical standard error, leading to slight overcoverage. With AIPTW, G-computation and TMLE, the model-based standard errors underestimate the empirical standard errors, leading to undercoverage and high type I error. Underestimation of the standard error based on the efficient influence function for the TMLE has been reported in the literature [28, 36]. The results for AIPTW and IPTW when there are a high number of covariates should be interpreted with caution as they lead to convergence issues; see Table S1 in Additional file 1 for more details. Results for $n=100$ are provided in Additional file 1, where we observe similar patterns, although the issues are alleviated at the larger sample size.

### Extension 2: Interaction

**Fig. 8:**
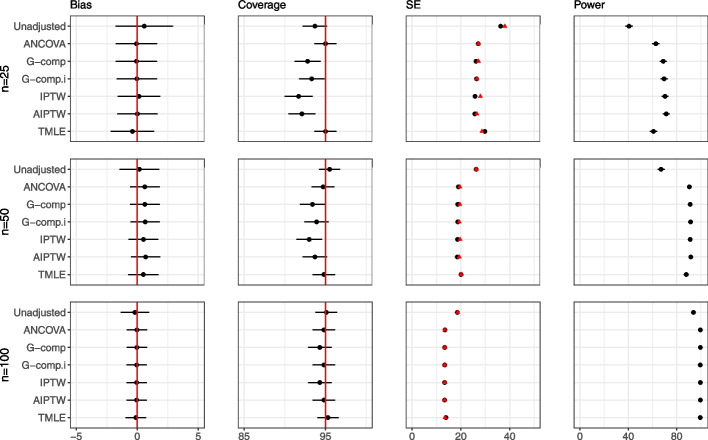
Extension 2 results (continuous outcome, single covariate, interaction) for the large interaction scenario. The performance of analytic methods in terms of bias, coverage and power is displayed. Model-based standard errors are shown in black and empirical standard errors are shown in red. Estimates are shown with $\pm 1.96 \times$ Monte Carlo standard error bars. Note that the scale of the bias is different for the four graphs, and the error bars are too small to be seen for power, due to the scale of the plots

**Fig. 9:**
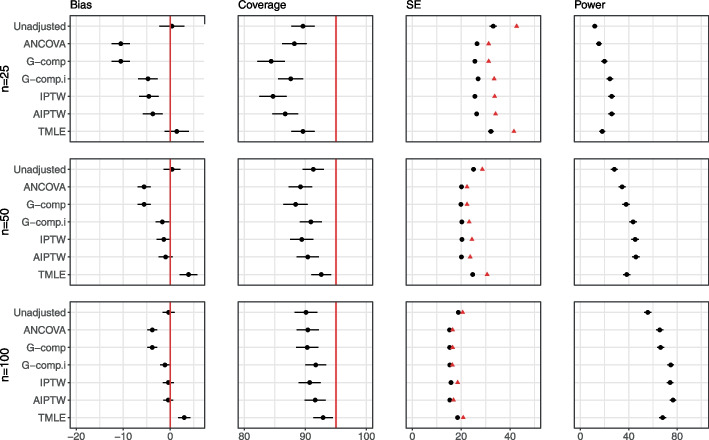
Extension 2 results (continuous outcome, single covariate, interaction) for the absent in one group scenario. The performance of analytic methods in terms of bias, coverage and power is displayed. Model-based standard errors are shown in black and empirical standard errors are shown in red. Estimates are shown with $\pm 1.96 \times$ Monte Carlo standard error bars. Note that the scale of the bias is different for the four graphs, and the error bars are too small to be seen for power, due to the scale of the plots

Figures 8 and 9 display the results for *large interaction* and *absent in one group* settings for Extension 2. Results for the *small interaction* and *different shapes* settings are shown in Additional file 1. For the *small interaction* and *large interaction* scenarios, all methods are unbiased, and standard errors are reduced as sample size increases. As before, the falsely small standard errors for IPTW, AIPTW and G-computation lead to undercoverage and false gains in power when sample size is small.

In the *Different shapes* and *Absent in one group* settings, the effect of the covariate is different in each arm. In the *Absent in one group* setting, the covariate is very skewed and is associated with the outcome only in one arm. In these settings, we see large biases with some methods, and biases are present even when sample size is 100. In particular, in the extreme setting of *Absent in one group* setting, we see that the biases are particularly pronounced for the ANCOVA, the spline and G-computation. It appears that G-computation with interaction, IPTW and AIPTW do not suffer from bias as much as G-computation without interaction and the regression-based methods. The undercoverage in the unadjusted approach in the *absent in one group* setting is due to unequal variances between the treatment and control groups; at sample size of 1000, the unadjusted approach achieves nominal coverage.

### Extension 3: Binary outcome

**Fig. 10:**
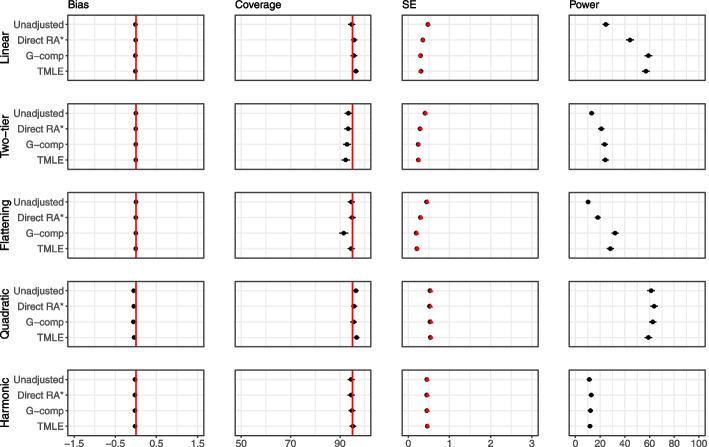
Extension 3 results (binary outcome, single covariate, no interaction) for sample size 100 and odds ratio as the estimand of interest. The performance of analytic methods in terms of bias, coverage and power is shown for the five different covariate–outcome relationships. Bias calculated for the marginal log-odds for all methods except for direct regression adjustment (RA). Model-based standard errors are shown in black and empirical standard errors are shown in red. The conditional odds ratio is 0.2. * For direct regression adjustment (RA), the bias is the difference between the estimated conditional odds ratio, and the data generating conditional odds ratio. The standard errors for direct RA cannot be compared to the other approaches

Figure 10 displays results for the binary outcome case with the odds ratio as the estimand of interest when sample size is 100. For all methods except direct RA, bias in the estimate of the marginal log odds ratio is shown. For direct RA, bias in the estimated conditional log odds ratio relative to the data generating conditional log odds ratio is shown. For all relationships, we observe that estimates are unbiased. Coverage appears to be reasonable for all methods. We observe that, as in the continuous outcome case, adjustment leads to gain in power when the covariate–outcome relationship can be approximated by a linear relationship. For the highly nonlinear *quadratic* and *harmonic* relationships, there are no gains in power.

**Fig. 11:**
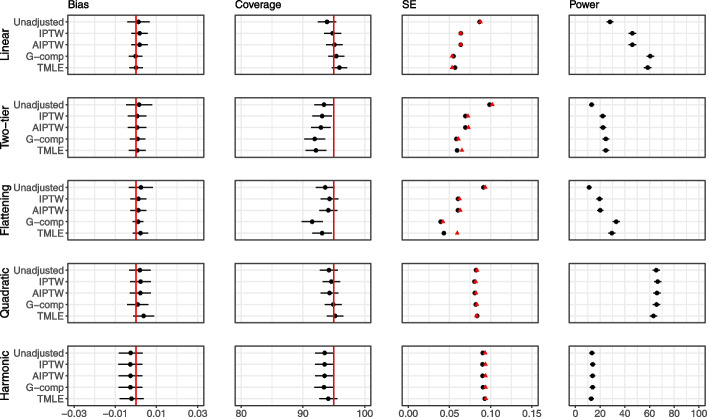
Extension 3 results (binary outcome, single covariate, no interaction) for sample size 100 and risk difference the estimand of interest. The performance of analytic methods in terms of bias, coverage and power are shown for the five different covariate–outcome relationships. Model-based standard errors are shown in black and empirical standard errors  are shown in red

Figure 11 displays results for the binary outcome case with the risk difference as the estimand of interest when sample size is 100. Convergence issues occur in the adjusted approach (in over $90\%$ of simulations for the *linear, two-tier, and flattening* relationships, over $60\%$ of simulation for the *quadratic* relationship, and over $3\%$ of simulations for the *harmonic* relationship), so these results are omitted. All other adjustment methods produce unbiased estimates. Coverage and type I error appear reasonable although there is evidence of slight undercoverage for nonlinear relationships; for the *two-tier* relationship, AIPTW, G-computation and TMLE lead to slight undercoverge, and for the *flattening* relationship, G-computation and TMLE lead to slight undercoverage. The standard error is particularly underestimated for TMLE. Similarly to the odds ratio, we observe that gains in power for adjustment are strongest when the covariate–outcome relationship is approximately linear.

## Discussion

Adjustment for baseline covariates in clinical trials have been shown to be beneficial where sample size is moderate to large. We investigated whether the benefits of adjustment—gain in power while estimates remain unbiased and coverage remains at the nominal level—are retained when there is potential for misspecification of the covariate–outcome relationship, and where sample size is small. We considered a wide range of adjustment methods including lesser-used methods such as IPTW, AIPTW, G-computation and TMLE, and considered whether they offer any advantages over the commonly used ANCOVA. See Table 1 for a summary of the analytic methods. We note that our simulations considered parallel design trial settings where randomization is 1:1 at the individual level, and our findings may not hold under other randomization schemes [37]. We also note that a number of settings explored in our simulations are unrealistic, but have been chosen as they may highlight areas where important differences between adjustment methods exist.

**Table 1:** Summary of analytic methods and their properties

Method	Properties
**Unadjusted**	Unbiased in all settings.
	Typically reduced power compared to adjusted approaches.
**ANCOVA/Adjusted**	Typically leads to increases in power.
	Retains good properties if many covariates adjusted for.
	No issues with estimation of standard errors in small samples.
	Bias in non-linear interaction setting.
	Marginal odds ratio cannot be targeted.
	Convergence issues if risk ratio is of interest.
**G-computation**	Undercoverage and high type I error in small sample sizes.
	Bias in non-linear interaction setting; alleviated by allowing for interaction.
**IPTW**	Covariate–outcome relationship need not be specified.
	Undercoverage and high type I error in small sample sizes and adjusting for a few covariates.
	Overcoverage if adjusting for many covariates.
	Convergence issues if there are many covariates.
	Slight bias in non-linear interaction setting.
**AIPTW**	Either covariate–treatment or covariate–outcome relationship needs to be correct.
	Undercoverage and high type I error in small sample sizes.
	Convergence issues if there are many covariates.
	Slight bias in non-linear interaction setting.
**TMLE**	Either covariate–treatment or covariate–outcome relationship needs to be correct.
	Standard errors can be underestimated if efficient influence function based estimators are used.
	Slight bias in non-linear interaction setting.

### Should you adjust?

Our simulations showed that, for the continuous outcome case where sample size is at least 100 and adjustment is for a few covariates, and there are no strong covariate–treatment interactions, all methods have desirable properties.

In the continuous outcome case, where there is non-linearity in the covariate–outcome relationship, methods that allow for non-linearities lead to greater gain in power than the common ANCOVA approach; these include the ANCOVA with spline, G-computation with interaction, G-computation with spline and IPTW. The recommendation by Kahan et al. [12] to use splines to model the covariate–outcome relationship is relevant in smaller trials.

When covariate–treatment interactions exist, adjustment methods can suffer from bias in small samples. The bias reduces with increasing sample size. If the interaction is non-linear and the covariate distribution is skewed, bias can present even in large samples. We prove this property and demonstrate it using simulation. Adjustment methods which allow for the presence of an interaction (including IPTW and G-computation with interaction) achieve an unbiased estimate at smaller sample sizes than the ANCOVA and other methods which do not allow for an interaction. If unexpected strong covariate–treatment interactions exist, bias can be induced by adjustment, particularly in small samples. However, if substantial interaction effects are suspected to exist a priori then the relevance of the marginal estimand is questionable; trial designs that explore treatment effect heterogeneity may be more appropriate.

For the binary outcome case, the treatment coefficient from an adjusted binomial model with a logistic link function does not target the marginal estimand. Further, small sample sizes are likely to lead to convergence issues. For the adjusted binomial model with linear link function, convergence issues are present even with $n=100$.

### Which covariates should be adjusted for?

While the focus of our simulation study was not in the selection of covariates for adjustment in the design/analysis stage of a trial, our simulations have shown that increase in power due to adjustment occurs when covariates are prognostic of outcome and the covariate–outcome relationship is linear or approximately linear, as shown previously by Kahan et al. [2]. If the relationship is non-linear but the adjustment approach allows for flexibility in the modelling of this relationship via splines, gains in power can also be achieved. However, the choice of degrees of freedom for the splines should be sensible relative to the sample size.

If a large number of covariates are adjusted for, we found that the statistical properties of ANCOVA were similar when the number of covariates was a select few. However, for other adjustment methods, adjusting for a high number of covariates led to over- or under-coverage and high type I error.

### Is it possible to improve on the ANCOVA?

We found that all methods performed well when sample sizes are moderate and covariate–treatment interactions are absent. We identified two settings where alternative adjustment methods to ANCOVA provide improvement. Firstly, where there are covariate–treatment interactions, G-computation with interaction, IPTW and AIPTW are potentially promising approaches. Secondly, our simulations showed that adding non-linearities by splines with a suitable number of degrees of freedom help to gain power when the true covariate–outcome relationship is non-linear. If the true relationship approximately linear, the addition of spline terms generally do not lead to loss of power.

In addition, for binary outcomes with the odds ratio as the estimand of interest, an advantage of using AIPTW, IPTW, G-computation and TMLE is that these adjustment approaches retain the marginal estimand, whereas regression-based approaches such as the ANCOVA and spline change the estimand.

### What areas need further investigation?

There are several possible extensions to our simulation study. Firstly, our study considered only linear interactions between treatment and covariate; future work could explore performance of adjustment methods in the presence of more complex interaction terms, which could potentially be misspecified. Secondly, while we attempted to provide a thorough exploration of this area, we did not explore all possible combinations of settings in our simulation study. In particular, we only explored the addition of spline terms in a selection of approaches (ANCOVA, G-computation and IPTW). We would expect similar improvements to be seen with the addition of splines to the AIPTW and TMLE approaches; further research could explore this. Thirdly, exploring the multiple covariates setting and the covariate–treatment interaction setting when the outcome is binary is a potential area of future work. Fourthly, improved performance might be achieved by other covariate adjustment methods, such as the recently-proposed overlap weights. Zeng et al. [38] demonstrated that overlap weights lead to improved precision compared to ANCOVA, IPTW and AIPTW when there is potential for model misspecification. Future work could consider this, and other approaches.

An issue that was identified in our simulation studies is that, for smaller sample sizes, G-computation, AIPTW and IPTW lead to underestimation of the standard error. Small sample corrections have been proposed, for instance, by Tsiatis et al. [4], and used in a trial setting by Van Lancker et al. [39]. The bias-corrected and accelerated (BCa) bootstrap has been shown to improve performance [40]. These small-sample corrections could be usefully evaluated in subsequent comparisons and incorporated into standard statistical software. Underestimation of standard errors can also occur for TMLE if efficient influence function based variance estimators are used; bootstrapping and stratified TMLE have been recommended as alternative approaches, which were not explored in this study [28, 36]. A further issue for TMLE is that, when using data-adaptive flexible models for the propensity score and outcome models, the estimated standard errors based on the efficient influence function are not doubly-robust, in the sense that their validity requires both models to be correct. While our simulation results indicate that the estimates of the standard errors are robust to model misspecification, we caution that this may not hold in general. Recent proposals give double robust inference for TMLE and other doubly-robust estimators in some settings [30, 41, 42]. This is an area for further research.

Lastly, the application of data-adaptive approaches for covariate adjustment is an emerging area of research; future work could consider these approaches. Williams et al. [40], for example, used machine learning approaches for variable selection to construct a model-robust, covariate-adjusted estimator for time-to-event and ordinal outcomes.

Practitioners can be reassured that covariate adjustment in settings commonly encountered in clinical trials generally leads to gains in power while estimates remain unbiased and coverage is at nominal level. The choice of method, ideally made in the planning stages of the trial, should take into account whether covariate–treatment interaction is likely and whether the sample size is sufficient for the use of methods that rely on large-sample properties.

### Supplementary information

**Additional file 1.**

**Additional file 2.**

**Additional file 3.**

**Additional file 4.**

## Data Availability

All data generated or analysed during this study are included in this published article and its supplementary information files.

## Appendix

We demonstrate that bias can be incurred for the ANCOVA when there is a covariate–treatment interaction, the covariate has a skewed distribution and the covariate–outcome relationship is misspecified.

For the simple ANCOVA, as in Eq. (5), the least squares/maximum likelihood parameter estimates of the effect of treatment $\beta _x$ and effect of covariate $\gamma$ are given by:

$$\begin{aligned} \hat{\beta }_x = (\overline{y}_1 - \overline{y}_0) - \hat{\gamma } (\overline{x}_1 - \overline{x}_0), \end{aligned}$$

$$\begin{aligned} \hat{\gamma } = \frac{\sum z_i (y_i - \overline{y}_1)(x_i - \overline{x}_1) + \sum (1 - z_i)(y_i - \overline{y}_0)(x_i - \overline{x}_0)}{\sum z_i (z_i - \overline{x}_1)^2 + \sum (1-z_i) (x_i - \overline{x}_0)^2}. \end{aligned}$$

We first show that $\hat{\gamma }$ can be expressed as an inverse-weighted average of the effect of the covariate for each arm. To do this, we fit a regression model for the effect of the covariate in each arm:

$$\begin{aligned} Y= & {} \alpha ^{(0)} + \gamma ^{(0)} X + \epsilon ^{(0)} \qquad \text {if} \qquad Z=0 \\ Y= & {} \alpha ^{(1)} + \gamma ^{(1)} X + \epsilon ^{(1)} \qquad \text {if} \qquad Z=1, \end{aligned}$$

where $$\epsilon ^{k} \sim N(0, \sigma ^2_{(k)})$$.

The least squares/maximum likelihood estimates for the effect of the covariate in each arm, $\gamma ^{(1)}$ and $\gamma ^{(0)}$ are given by:

$$\begin{aligned} \hat{\gamma }^{(1)}= & {} \frac{\sum z_i (y_i - \overline{y}_1)(x_i - \overline{x}_1)}{\sum z_i (x_i - \overline{x}_1)^2} \\ \hat{\gamma }^{(0)}= & {} \frac{\sum (1 - z_i)(y_i - \overline{y}_0)(x_i - \overline{x}_0)}{\sum (1-z_i) (x_i - \overline{x}_0)^2} \end{aligned}$$

Further, the estimates of their variances are given by:

$$\begin{aligned} Var(\hat{\gamma }^{(1)})= & {} \frac{\sigma ^2_{(k)} }{\sum z_i (x_i - \overline{x}_1)^2}, \\ Var(\hat{\gamma }^{(0)})= & {} \frac{\sigma ^2_{(k)} }{\sum (1-z_i) (x_i - \overline{x}_0)^2}. \end{aligned}$$

We assume that $\sigma _{(1)}^2 = \sigma _{(0)}^2$ and define inverse variance weights $w_0$ and $w_1$:

$$\begin{aligned} w_1= & {} (\sigma ^2)^{(-1)} \sum z_i (x_i - \overline{x}_1)^2, \\ w_0= & {} (\sigma ^2)^{(-1)} \sum (1-z_i) (x_i- \overline{x}_0)^2 \end{aligned}$$

We can then write the the ANCOVA estimate of the treatment effect as an inverse-weighted average of the slopes within each arm:

$$\begin{aligned} \hat{\gamma } = \frac{w_1 \hat{\gamma }^{(1)} + w_0 \hat{\gamma }^{(0)}}{w_1 + w_0}. \end{aligned}$$

We can then write the effect of treatment as follows:

$$\begin{aligned} \hat{\beta }_x= & {} (\overline{y}_1 - \overline{y}_0) - \hat{\gamma } (\overline{x}_1 - \overline{x}_0), \\= & {} (\overline{y}_1 - \overline{y}_0) - \frac{w_1 \hat{\gamma }^{(1)} + w_0 \hat{\gamma }^{(0)}}{w_1 + w_0}(\overline{x}_1 - \overline{x}_0). \end{aligned}$$

The weights $w_1$ and $w_0$ tend in probability to 0.5. To simplify our argument below, we replace the weights with these limits. Thus, we have that the expectation of the marginal estimator $\hat{\beta }$ is:

12 $$\begin{aligned} \mathbb {E}\left[ \hat{\beta } \right]= & {} \mathbb {E}\left[ \overline{y}_1 \right] - \mathbb {E}\left[ \overline{y}_0\right] - \mathbb {E}\left[ \frac{w_1 \hat{\gamma }^{(1)} + w_0 \hat{\gamma }^{(0)}}{w_1 + w_0}(\overline{x}_1 - \overline{x}_0) \right] \nonumber \\= & {} \beta - \mathbb {E} \left[ \left( {0.5 \hat{\gamma }^{(1)} + 0.5 \hat{\gamma }^{(0)}} \right) (\overline{x}_1 - \overline{x}_0) \right] \nonumber \\= & {} \beta - 0.5\mathbb {E}\left[ \hat{\gamma }^{(1)}\overline{x}_1 \right] + 0.5 \mathbb {E}\left[ \hat{\gamma }^{(1)}\overline{x}_0\right] - 0.5 \mathbb {E}\left[ \hat{\gamma }^{(0)}\overline{x}_1\right] \nonumber \\&+ 0.5 \mathbb {E}\left[ \hat{\gamma }^{(0)}\overline{x}_0\right] \nonumber \\= & {} \beta - 0.5 \mathbb {E}\left[ \hat{\gamma }^{(1)} \overline{x}_1\right] +0.5 \mathbb {E}\left[ \hat{\gamma }^{(1)}\right] \mathbb {E} \left[ \overline{x}_0\right] - 0.5 \mathbb {E}\left[ \hat{\gamma }^{(0)}\right] \mathbb {E}\left[ \overline{x}_1\right] \end{aligned}$$

13 $$\begin{aligned}&+ 0.5 \mathbb {E}\left[ \hat{\gamma }^{(0)}\overline{x}_0\right] \nonumber \\= & {} \beta - 0.5 \mathbb {E}\left[ \hat{\gamma }^{(1)} \overline{x}_1\right] +0.5 \gamma ^{(1)} \mathbb {E} \left[ \overline{x}_0\right] - 0.5 {\gamma }^{(0)}\mathbb {E}\left[ \overline{x}_1\right] \end{aligned}$$

14 $$\begin{aligned}&+ 0.5 \mathbb {E}\left[ \hat{\gamma }^{(0)}\overline{x}_0\right] \nonumber \\= & {} \beta - 0.5 \mathbb {E}\left[ \hat{\gamma }^{(1)} \overline{x}_1\right] +0.5 \gamma ^{(1)} \mathbb {E} \left[ \overline{x}_1\right] - 0.5 {\gamma }^{(0)}\mathbb {E} \left[ \overline{x}_1\right] \end{aligned}$$

15 $$\begin{aligned}&+ 0.5 \mathbb {E}\left[ \hat{\gamma }^{(0)}\overline{x}_0\right] \nonumber \\= & {} \beta - 0.5 \left[ \text {Cov}(\hat{\gamma }^{(1)} \overline{x}_1 ) - \text {Cov}(\hat{\gamma }^{(0)}, \overline{x}_0 ) \right] \end{aligned}$$

where Eq. (12) follows since we have that $\hat{\gamma }^{(1)}$ is independent of $\overline{x}_0$, and $\hat{\gamma }^{(0)}$ is independent of $\overline{x}_1$. Equation (13) follows since $\mathbb {E}\left[ \hat{\gamma }^{(1)} \right] = \gamma ^{(1)}$, and $\mathbb {E}\left[ \hat{\gamma }^{(0)} \right] = \gamma ^{(0)}$. Equation (14) follows since for a randomized experiment, $\mathbb {E} \left[ \overline{x}_1 \right] = \mathbb {E} \left[ \overline{x}_0 \right]$.

The bias due to adjustment by ANCOVA in a trial setting with 1:1 randomisation can therefore be written as $0.5 \left[ \text {Cov}(\hat{\gamma }^{(1)}, \overline{x}_1 ) - \text {Cov}(\hat{\gamma }^{(0)}, \overline{x}_0 ) \right]$. This bias is therefore approximately zero in expectation for sufficiently large sample sizes. For finite samples, if the effect of the treatment is different in each arm, the covariate has skewness, and further, the ANCOVA leads to misspecification of the covariate–outcome relationship, this bias may be particularly pronounced.

## Abbreviations

AIPTW: Augmented inverse probability of treatment weighting
ANCOVA: Analysis of covariance
Direct RA: Direct regression adjustment
IPTW: Inverse probability of treatment weighting
OR: Odds ratio
TMLE: Targeted maximum likelihood estimation
