# Supplementary material for: A comparison of covariate adjustment approaches under model misspecification in individually randomized trials
Source: Trials. 2023 Jan 6;24:14. doi: 10.1186/s13063-022-06967-6 (PMC9817411; doi:10.1186/s13063-022-06967-6)
Supplement: Supplementary file 1 — Additional file 1. [file 13063_2022_6967_MOESM1_ESM.pdf]

A Comparison of Covariate Adjustment Approaches under Model Misspecification in Individually Randomized Trials: Additional File 1

1 Data Generating Mechanisms

1.1 Main Simulation

We assume a baseline risk factor $x_i \sim N(0,1)$ and error term $e_i \sim N(0, 60^2)$ for all i. Outcomes are generated under each covariate-outcome relationship as follows:

- *Linear* relationship:

$$y_i^0 = 395 + 110x_i + e_i$$
$$y_i^1 = 395 + 110x_i + 40 + e_i.$$

- *Two-tier* relationship:

$$y_i^0 = 180 + 470\mathbb{I}(x_i > 0) + e_i$$
$$y_i^1 = 180 + 470\mathbb{I}(x_i > 0) + 40 + e_i,$$

  where $\mathbb{I}(\cdot)$ denotes the indicator function.

- *Flattening* relationship:

$$y_i^0 = 700 - \exp(-x_i + 4) + e_i$$
$$y_i^1 = 700 - \exp(-x_i + 4) + 40 + e_i.$$

- *Quadratic* relationship:

$$y_i^0 = 100 + 104x_i^2 + e_i$$
$$y_i^1 = 100 + 104x_i^2 + 40 + e_i.$$

- *Harmonic* relationship:

$$y_i^0 = 400 + 300\cos(0.9\pi x_i + 4) + e_i$$
$$y_i^1 = 400 + 300\cos(0.9\pi x_i + 4) + 40 + e_i.$$

1.2 Extension 1: Multiple covariates

Variables $\boldsymbol{x}_1, \boldsymbol{x}_2, ..., \boldsymbol{x}_{10}$ are generated from a multivariate normal distribution with mean given by a vector of zeros and correlation given by R:

$$R = \begin{pmatrix} 1 & -0.158 & 0.02 & -0.336 & -0.028 & -0.029 & 0.125 & 0.073 & -0.008 & -0.052 \\ -0.158 & 1 & 0.215 & 0.023 & -0.239 & -0.241 & 0.289 & -0.426 & -0.187 & 0.255 \\ 0.02 & 0.215 & 1 & 0.017 & -0.036 & -0.094 & 0.076 & -0.096 & -0.011 & 0.094 \\ -0.336 & 0.023 & 0.017 & 1 & -0.168 & -0.111 & 0.123 & -0.312 & -0.099 & 0.095 \\ -0.028 & -0.239 & -0.036 & -0.168 & 1 & 0.395 & -0.477 & 0.461 & 0.306 & -0.084 \\ -0.029 & -0.241 & -0.094 & -0.111 & 0.395 & 1 & -0.493 & 0.294 & 0.132 & -0.063 \\ 0.125 & 0.289 & 0.076 & 0.123 & -0.477 & -0.493 & 1 & -0.609 & -0.338 & 0.143 \\ 0.073 & -0.426 & -0.096 & -0.312 & 0.461 & 0.294 & -0.609 & 1 & 0.472 & -0.332 \\ -0.008 & -0.187 & -0.011 & -0.099 & 0.306 & 0.132 & -0.338 & 0.472 & 1 & -0.34 \\ -0.052 & 0.255 & 0.094 & 0.095 & -0.084 & -0.063 & 0.143 & -0.332 & -0.34 & 1 \end{pmatrix}.$$

From these variables, the following 21 continuous covariates are generated:

1. Continuous, mimics age: $c_{1,i} = x_{1,i} \cdot 14.3 + 48.9$
2. Continuous, mimics BMI: $c_{2,i} = 1/(0.007x_{2,i} + 0.0377)$
3. Continuous, mimics baseline vitamin D: $c_{3,i} = (1.756x_{3,i} + 7.167)^2 - 5$
4. Continuous, mimics baseline PEFR: $c_{4,i} = 109.75x_{4,i} + 379.8$
5. Continuous, mimics baseline Asthma score: $c_{5,i} \sim \text{Exp}(\Phi(x_{5,i}), \lambda = 2)$
6. Continuous, mimic baseline Vent score: $c_{6,i} \sim \text{Exp}(\Phi(x_{6,i}), \lambda = 1)$
7. Continuous, mimics baseline ACT score: $c_{7,i} = 4.5d_7 + 20$, where $d_7 \sim \mathcal{N}\left(0.867 \times \Phi(x_{7,i})\right)$
8. Continuous, mimics baseline SGRQ score $c_{8,i} = (1.59x_{8,i} + 4.75)^2$
9. Continuous, mimics baseline RQLQ score $c_{9,i} = (0.512x_{9,i} \cdot +1.036)^2$
10. Continuous, mimics baseline EuroQoL score $c_{10,i} = 20d_{10,i} + 80$, where $d_{10,i} \sim \mathcal{N}\left(0.841 \times \Phi(x_{10,i})\right)$
11. Binary, mimics sex: $c_{11,i} \sim \text{Bernoulli}(p_{11,i})$, where $p_{11,i} = 8.36 - 0.196c_{1,i} - 0.650c_{2,i} + 0.0095c_{1,i}c_{2,i} - 0.009c_{3,i} + 0.020c_{4,i} + 0.568c_{5,i} - 0.023c_{6,i} + 0.015c_{7,i} + 0.0007c_{8,i} - 0.364c_{9,i} - 0.022c_{10,i}$

12. $c_{12,i} \sim \text{Bernoulli}(p_{11,i})$, where
$p_{11,i} = -17.45 + 28.29c_{11,i} + 0.203c_{1,i} + 0.279c_{2,i} - 0.0063c_{1,i}c_{2,i} + 0.026c_{3,i} + 0.009c_{4,i} - 0.353c_{5,i} + 0.210c_{6,i} + 0.234c_{7,i} + 0.021c_{8,i} - 0.168c_{9,i} + 0.020c_{10,i} - 0.4538531c_{1,i}c_{11,i} - 0.894c_{2,i}c_{11,i} + 0.0174c_{1,i}c_{2,i}c_{11,i} + 0.020c_{3,i}c_{11,i} - 0.006c_{4,i}c_{11,i} + 2.875c_{5,i}c_{11,i} + 0.046c_{6,i}c_{11,i} - 0.159c_{7,i}c_{11,i} - 0.072c_{8,i}c_{11,i} + 0.415c_{9,i}c_{11,i} - 0.022c_{10,i}c_{11,i}$

13. $c_{13,i} \sim \text{Bernoulli}(p_{13,i})$, where
$p_{13,i} = -3.61 - 1.829c_{11,i} + 0.016c_{1,i} + 0.245c_{2,i} + 0.04c_{3,i} + 0.001c_{4,i} - 0.426c_{5,i} - 0.276c_{6,i} - 0.044c_{7,i} - 0.079c_{8,i} + 0.849c_{9,i} - 0.040c_{10,i}$

14. Binary, mimics smoking: $c_{14,i} \sim \text{Bernoulli}(p_{14,i})$ where
$p_{14,i} = -6.23 + 1.13c_{12,i} - 0.381c_{13,i} - 0.032c_{11,i} + 0.342c_{1,i} - 0.0045c_{1,i}^2 - 0.218c_{2,i} + 0.012c_{3,i} - 0.002c_{4,i} - 0.734c_{5,i} + 0.77c_{6,i} + 0.044c_{7,i} + 0.051c_{8,i} - 0.659c_{9,i} + 0.021 \cdot c_{10,i}$

15. Continuous, collinear noise variable: $c_{15,i} \sim \text{Exp}(\Phi(u_{15,i}), \lambda = 2)$, where
$u_{15,i} = \sqrt{0.95}x_{4,i} + \sqrt{0.05}Z$, where $Z \sim N(0,1)$.

16. Continuous, collinear noise variable: $c_{16,i} \sim 10 \times \mathcal{N}(0.841 \times \Phi(u_{16,i})) + 40$,
where $u_{16,i} = \sqrt{0.95}x_{4,i} + \sqrt{0.95}Z$

17. Continuous, collinear noise variable: $c_{17,i} \sim \chi^2(\Phi(u_{17,i}), 4)$, where
$u_{17,i} = \sqrt{0.95}x_{4,i} + \sqrt{0.05}Z$

18. Continuous, noise variable: $c_{18,i} \sim N(0,1)$

19. Continuous, noise variable: $c_{19,i} \sim \text{Exp}(\Phi(Z), \lambda = 2)$

20. Continuous, noise variable:$c_{20,i} \sim 10 \times \mathcal{N}(0.841 \times \Phi(Z)) + 40$

21. Continuous, noise variable: $c_{21,i} = \chi(\Phi(Z), 4)$.

The covariates $\boldsymbol{c}_1, \boldsymbol{c}_2, ..., \boldsymbol{c}_{21}$ are centred and standardized, and denoted $\boldsymbol{d}_1, \boldsymbol{d}_2, ..., \boldsymbol{d}_{21}$.

Outcomes under the control arm are given below, where $V \sim N(0, 39.4^2)$:

$$
\begin{aligned}
y_i^0 = {} & 381.1555 - 9.226248d_{14,i} + 12.07d_{12,i} + 23.29d_{13,i} + 1.525504d_{11,i} - 10.4878d_{1,i} \\
& - 0.595686d_{2,i} - 0.1443187d_{1,i}d_{2,i} + 0.0335872d_{3,i} + 103.1218d_{4,i} - 4.413275d_5 \\
& - 1.633389d_{6,i} + 1.02916d_{7,i} + 4.685524d_{8,i} + 1.990803d_{9,i} + 8.379155d_{10,i} \\
& + 16.73711d_{1,i}d_{11,i} - 4.196649d_{2,i}d_{11,i} + 26.74518d_{1,i}d_{2,i}d_{11,i} - 4.499786d_{3,i}d_{11,i} \\
& + 9.622228d_{4,i}d_{11,i} + 2.60351d_{5,i}d_{11,i} + 7.98627d_{6,i}d_{11,i} + 9.375551d_{7,i}d_{11,i} \\
& - 14.80215d_{8,i}d_{11,i} + 5.965411d_{9,i}d_{11,i} - 20.99641d_{10,i}d_{11,i} + V
\end{aligned}
$$

Outcomes under the active arm are given by:

$$y_i^1 = y_i^0 + 42.$$

1.3 Extension 2: Interaction

We assume a baseline risk factor $x_i \sim N(0,1)$ and error term $e_i \sim N(0, 60^2)$ for all i. Outcome under each interaction setting are generated as follows:

- *Small Interaction* setting:

$$y_i^0 = 395 + 80x_i + e_i$$
$$y_i^1 = 395 + 80x_i + 20x_i + 40 + e_i.$$

- *Large Interaction* setting:

$$y_i^0 = 500 + 20x_i + e_i$$
$$y_i^1 = 500 + 20x_i + 90x_i - 65 + e_i.$$

- *Different Shapes* setting:

$$y_i^0 = 450 + 100x_i + e_i$$
$$y_i^1 = 450 + 100 - \exp(-(x_i - 3.5)) + e_i.$$

- *Absent in one group* setting:

$$y_i^0 = e_i$$
$$y_i^1 = 13x_i^4 + e_i.$$

1.4 Extension 3: Binary outcome

We assume a baseline risk factor $x_i \sim N(0,1)$ for all i. Outcomes on the logit scale for each covariate-outcome relationship are generated as follows:

- *Linear* relationship:

$$p_i^0 = -2 - 4x_i$$
$$p_i^1 = -2 - 4x_i + log(0.2).$$

- *Two-tier* relationship:

$$p_i^0 = -2 + 6\mathbb{I}(x_i > 0)$$
$$p_i^1 = -2 + 6\mathbb{I}(x_i > 0) + log(0.2).$$

- *Flattening* relationship:

$$p_i^0 = 10 - 15\exp(-x_i)$$
$$p_i^1 = 10 - 15\exp(-x_i) + log(0.2).$$

- *Quadratic* relationship:

$$p_i^0 = -4x_i^2 - 3.5x_i + 0.5$$
$$p_i^1 = -4x_i^2 - 3.5x_i + 0.5 + log(0.2)$$

- *Harmonic* relationship:

$$p_i^0 = -5 + 10\cos(0.9\pi x_i + 5.25)$$
$$p_i^1 = -5 + 10\cos(0.9\pi x_i + 5.25) + log(0.2).$$

Binary outcomes are obtained by setting:

$$y_i^0 = \begin{cases} 1 & \text{if } u_i \geq \text{expit}(p_i^0) \\ 0 & \text{if } u_i < \text{expit}(p_i^0) \end{cases}$$
$$y_i^1 = \begin{cases} 1 & \text{if } u_i \geq \text{expit}(p_i^1) \\ 0 & \text{if } u_i < \text{expit}(p_i^1) \end{cases},$$

where $u_i \sim \text{Unif}(0, 1)$ and $\text{expit}(\cdot)$ denotes the inverse-logit function.

2 Further results

2.1 Main Simulation

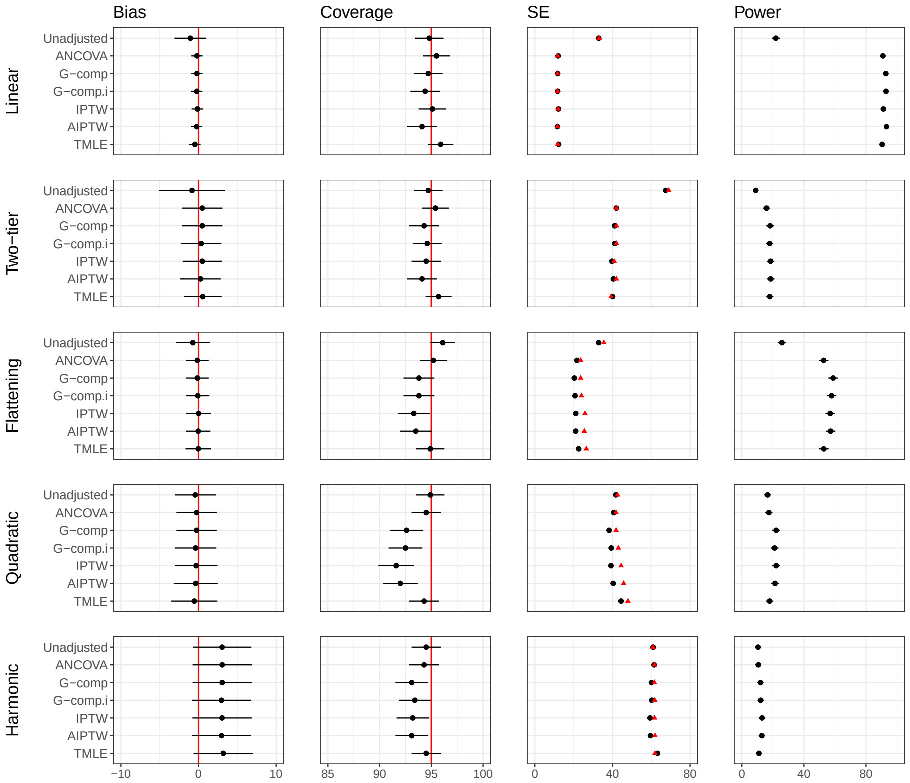

Figure 1: Main Simulation results for sample size 50. The performance of analytic methods in terms of bias, coverage and power for the five different covariate–outcome relationships are displayed. The effect of treatment is 40. Model-based standard errors are indicated in black and the empirical standard error is shown in red. Estimates are indicated with $\pm 1.96\times$ Monte Carlo standard error bars. Note that the error bars are too small to be seen for power, due to the scale of the plots.

In Figure 2 we observe apparent bias in the *two-tier* setting for the Main Simulation with $n = 100$. This is purely due to chance, as the results are unbiased when the number of repetitions is increased from 1000 to 5000, as shown in Figure 4 in the main text.

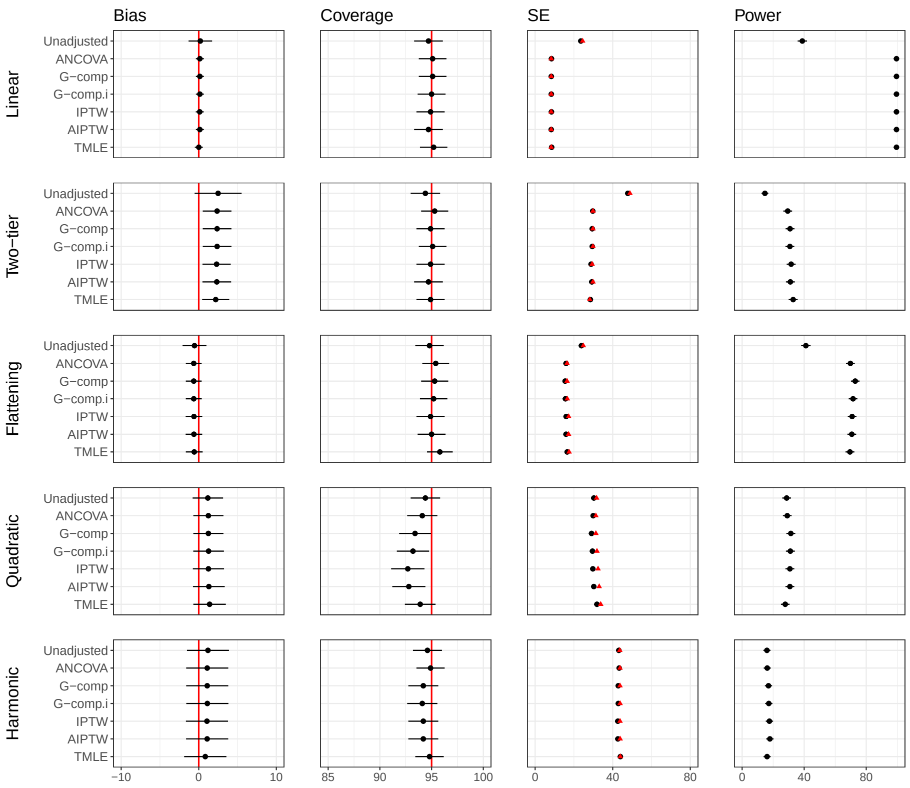

Figure 2: Main Simulation results for sample size 100. The number of repetitions for this simulation is 1000. The performance of analytic methods in terms of bias, coverage and power for the five different covariate–outcome relationships are displayed. The effect of treatment is 40. Model-based standard errors are indicated in black and the empirical standard error is shown in red. Estimates are indicated with $\pm 1.96\times$ Monte Carlo standard error bars. Note that the error bars are too small to be seen for power, due to the scale of the plots.

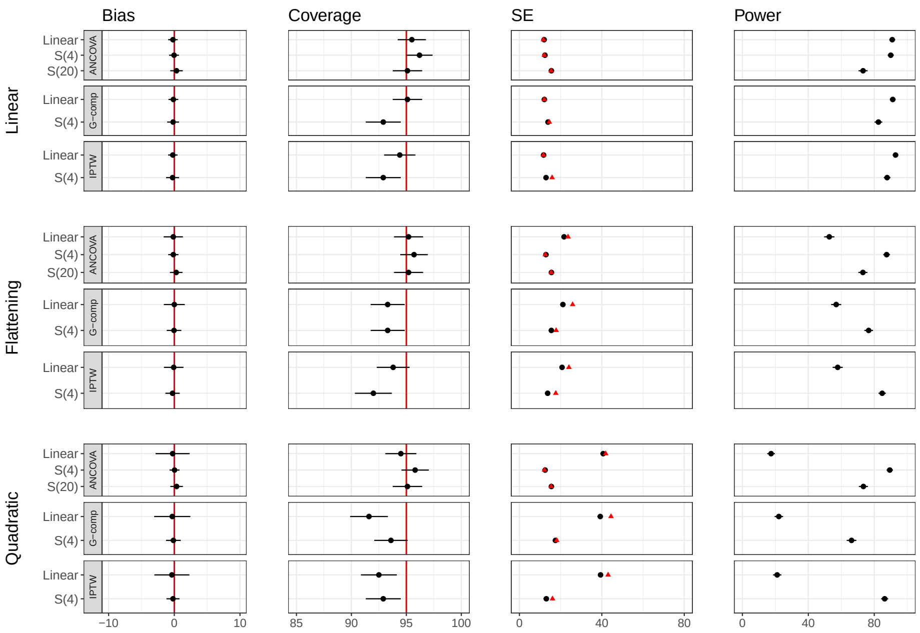

Figure 3: Main Simulation results for sample size 50 for ANCOVA, G-computation and IPTW with and without the use of splines. The performance of analytic methods in terms of bias, coverage and power for the *Linear, Flattening* and *Quadratic* relationships are displayedThe effect of treatment is 40. Model-based standard errors are indicated in black and the empirical standard error is indicated in red. Estimates are shown with $\pm 1.96\times$ Monte Carlo standard error bars. Note that the error bars are too small to be seen for power, due to the scale of the plots.

2.2 Extension 1: Multiple covariates

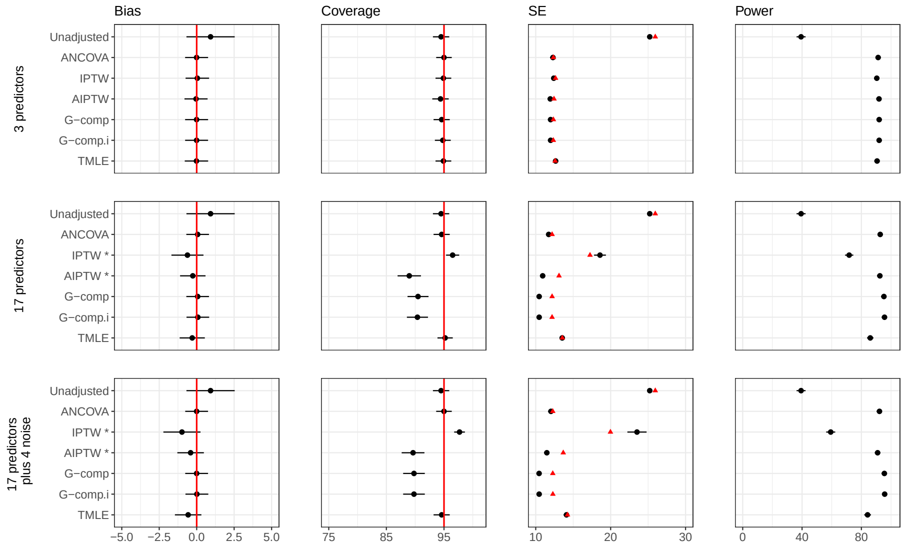

Figure 4: Extension 1 (multiple covariates) results for sample size 100. The performance of analytic methods in terms of bias, coverage and power are shown when there are 3 covariates, 17 covariates and 17 covariates plus 4 noise variables. The effect of treatment is 40. Model-based standard errors are shown in black and the empirical standard error is shown in red. Estimates are shown with ±1.96× Monte Carlo standard error bars. Note: the error bars are too small to be seen for power, due to the scale of the plots.
* AIPTW and IPTW suffer from convergence issues when there are a high number of predictors; see Table 1 for more details

Table 1: Details of simulation settings where the proportion of simulations that do not converge exceed 0.05

**Simulation**	**Setting**	n	**Estimand**	**Method**	**Proportion of simulations with non-convergence**
Extension 1: Multiple covariates	17 covariates	50	ATE=42	IPTW	0.07
	17 covariates	50	ATE=42	AIPW	0.56
	17 covariates	50	ATE=0	IPTW	0.07
	17 covariates	50	ATE=0	AIPW	0.56
	17 predictors + 4 noise	50	ATE=42	IPTW	0.187
	17 predictors + 4 noise	50	ATE=42	AIPW	0.619
	17 predictors + 4 noise	50	ATE=0	IPTW	0.187
	17 predictors + 4 noise	50	ATE=0	AIPW	0.619
	17 covariates	100	ATE=0	AIPTW	0.084
	17 predictors + 4 noise	100	ATE=42	AIPTW	0.084
	17 predictors + 4 noise	100	ATE=0	AIPTW	0.084

2.3 Extension 2: Interaction

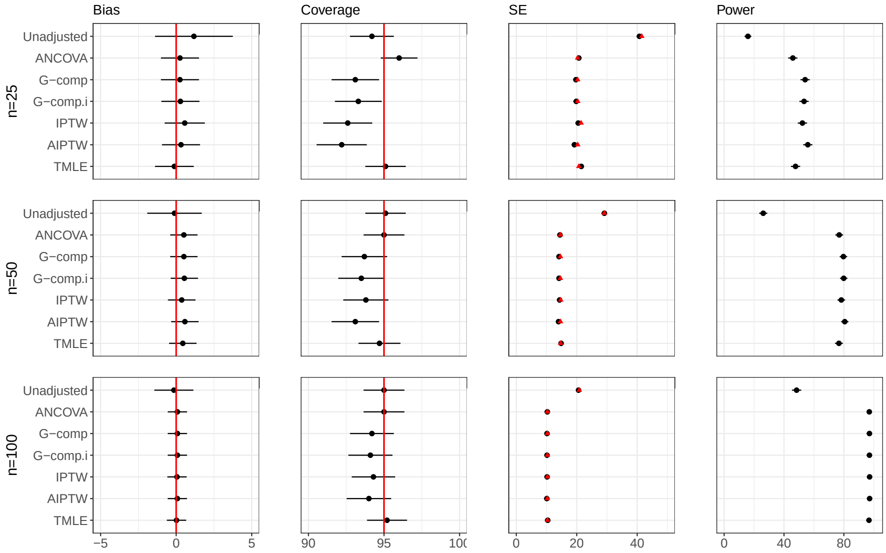

Figure 5: Extension 2 (Interaction) results for the small interaction scenario. The performance of analytic methods in terms of bias, coverage and power are displayed. Model-based standard errors are shown in black and the empirical standard error is shown in red. Estimates are shown with $\pm 1.96\times$ Monte Carlo standard error bars. Note that the scale of the bias is different for the four graphs, and the error bars are too small to be seen for power, due to the scale of the plots.

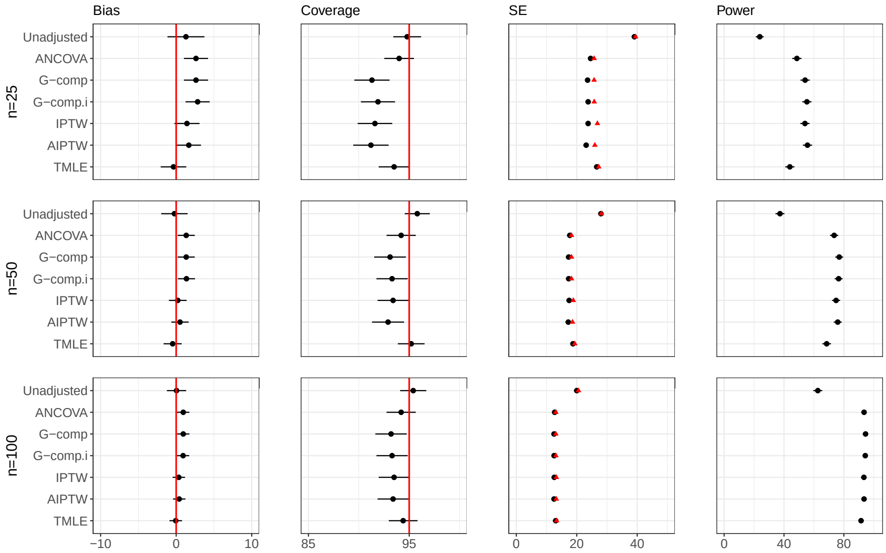

Figure 6: Extension 2 (Interaction) results for the different shapes scenario. The performance of analytic methods in terms of bias, coverage and power are displayed. Model-based standard errors are shown in black and the empirical standard error is shown in red. Estimates are shown with $\pm 1.96\times$ Monte Carlo standard error bars. Note that the scale of the bias is different for the four graphs, and the error bars are too small to be seen for power, due to the scale of the plots.
